# Evans Syndrome in a Jehovah’s Witness

**DOI:** 10.7759/cureus.15508

**Published:** 2021-06-07

**Authors:** Lex P Leonhardt, Aamir Pervez, Alejandro Calvo

**Affiliations:** 1 Internal Medicine, Kettering Medical Center, Dayton, USA; 2 Hematology and Medical Oncology, Kettering Medical Center, Dayton, USA

**Keywords:** evans syndrome, jehovah's witness, thrombocytopenia, microangiopathic autoimmune hemolytic anemia, transfusion refusal, blood dyscrasia, idiopathic thrombocytopenia

## Abstract

Evans syndrome (ES) is a rare hematologic disorder characterized by the development of autoimmune hemolytic anemia (AIHA), idiopathic thrombocytopenia, and occasionally immune-mediated neutropenia. Jehovah’s Witnesses (JW) often decline blood product transfusion on the grounds of a scriptural stand based on biblical texts. The acute management of ES often consists of blood product transfusion in addition to high-dose steroids and intravenous immunoglobulin. We describe the case of a JW female presenting with new-onset, acutely worsening AIHA and thrombocytopenia with concern for hemodynamic compromise who was successfully treated with erythropoietin-stimulating agents, parenteral iron, folic acid, and high-dose steroids.

## Introduction

Evans syndrome (ES) is a rare hematologic disorder characterized by the development of autoimmune hemolytic anemia (AIHA), idiopathic thrombocytopenia (ITP), and occasionally immune-mediated neutropenia. Secondary ES is usually associated with a wide array of underlying disorders, including systemic lupus erythematosus (SLE), autoimmune hepatitis, chronic lymphocytic leukemia, and autoimmune lymphoproliferative syndrome (ALPS) [1,2]. The exact prevalence of ES is unknown; however, rough estimates note one to nine persons per million based on small retrospective cohorts and observational studies [3]. A retrospective review in Denmark identified 242 patients diagnosed with ES from 1977 to 2017, noting a five-year mortality rate of 38% and a median survival of 1.7 years in patients with secondary ES specifically [4].

The acute management of ES often consists of blood product transfusion in addition to high-dose steroids and intravenous immunoglobulin (IVIG). Second-line therapies include rituximab, splenectomy, various immunosuppressants, and hematopoietic stem cell transplantation. The use of erythropoietin-stimulating agents (ESAs) and thrombopoietin receptor agonists (TPO-RA) has been rarely reported. The efficacy of second-line therapies varies on a case-to-case basis, attributed to the rarity of ES and lack of treatment recommendations. Rituximab has been shown to have a positive response in isolated AIHA and primary ES. While data are limited, there are several case reports that demonstrate a positive response to rituximab in patients with secondary ES and concomitant SLE. Immunosuppressants have similar variable outcomes and are often used in the setting of failed treatment with steroids, IVIG, rituximab, and splenectomy. The use of ESAs has previously been described in a multicentric retrospective international study in 51 patients diagnosed with isolated AIHA unresponsive to first-line therapies, with a mean increase in hemoglobin of 2 g/dL in 70% of cases after two and a half weeks of therapy [1]. However, the use of ESAs in ES specifically is limited to case reports and small case series with inconclusive results. Both TPO-RA and ESAs have been associated with thrombotic events when used in isolated AIHA [1,2].

Jehovah’s Witnesses (JW) often decline blood product transfusion on the grounds of a scriptural stand based on biblical texts. Practicing JW patients vary in their choice regarding blood product transfusion and their preference should be established on an individual basis [5]. We describe the case of a JW female presenting with new-onset, acutely worsening AIHA and thrombocytopenia with concern for hemodynamic compromise who was successfully treated with ESAs, parenteral iron, and folic acid, in addition to high-dose steroids. The importance of this case highlights the treatment of ES in the unusual circumstance in which blood products are refused despite acutely worsening and potentially life-threatening anemia and thrombocytopenia.

## Case presentation

A 40-year-old African American female with a past medical history of obesity and notable family history of SLE presented to the emergency department complaining of progressively worsening dyspnea, fatigue, generalized weakness, hematuria, and upper extremity petechial rash for one week. Vital signs were notable for tachycardia with a heart rate of 103 beats per minute, blood pressure of 103/75 mmHg, oxygen saturation of 94% on room air. On physical examination, pallor was noted with multiple areas of petechiae over bilateral proximal upper extremities. Initial hematologic workup was notable for hemoglobin of 5.4 g/dL, platelet count 51,000/µL, lactate dehydrogenase 575 U/L, undetectable haptoglobin (<8 mg/dL), total bilirubin 4.3 mg/dL with indirect predominance, and absolute reticulocyte count of 322 K/uL. At this point, our team developed a broad differential diagnosis including ALPS, thrombotic thrombocytopenic purpura, hemolytic uremic syndrome, common variable immunodeficiency syndrome, and antiphospholipid syndrome. Given abnormal hematologic parameters consistent with hemolytic anemia and thrombocytopenia, confirmatory direct antigen testing (DAT) immunoglobulin G (IgG) and DAT C3 were ordered and subsequently found to be positive. CT of the chest with intravenous contrast was ordered to rule out additional lymphoproliferative disorders and was unremarkable.

**Table 1:** Hematologic parameters on admission compared to hospital day four and fourteen days after discharge. MCV: mean corpuscular volume; MCH: mean corpuscular hemoglobin; MCHC: mean corpuscular hemoglobin concentration; RDW: red cell distribution width; LDH: lactate dehydrogenase

Test	At presentation	Hospital day 4	14 days post-discharge	Reference
White blood cells	7.7	14.5	14.3	4.0-10.5 K/µL
Red blood cells	1.37	2.14	3.42	3.86-5.17 M/µL
Hemoglobin	5.4	7.8	11.6	12.1-15.8 g/dL
Hematocrit	15.8	23.7	35.2	35.8-46.5%
MCV	126.8	110.9	103.2	85-99 fl
MCH	43.7	36.4	34.1	28.4-33.4 pg
MCHC	34.4	36.4	33.0	31.1-37.0 g/dL
RDW	20.2	19.2	16.0	11.7-15.2%
Platelet count	51	87	163	154-393 K/µL
Absolute neutrophil count	5.3	9.7	13.8	2.0-7.3 K/µL
Absolute lymphocytes	1.9	3.8	0.3	0.8-3.6 K/µL
Absolute monocytes	0.4	0.8	0.2	0.3-0.9 K/µL
Absolute eosinophils	0.1	0.1	0.0	0.0-0.4 K/µL
Absolute basophils	0.0	0.1	0.0	0.0-0.1 K/µL
LDH	575	472	286	84-286 U/L
Absolute reticulocyte	322.22	420.74	122.7	20.00-80.00 K/µL
Haptoglobin	<8	<8	45	30-200 mg/dL

Further hemodynamic instability was noted with worsening sinus tachycardia, heart rate reaching 120 bpm, and blood pressure dropping to 80/60 mmHg. Due to her religious beliefs, the patient refused all blood products despite a markedly decreased hemoglobin level associated with hemodynamic compromise. At this point, the decision was made to administer 40,000 units of erythropoietin, 3 mg oral folic acid daily, and intravenous iron sucrose 200 mg daily for two doses. The patient reported a questionable allergy to prednisone in which she developed diffuse hives and moderate shortness of breath several years prior despite tolerating Kenalog injections in the past. Given her history, intravenous methylprednisolone 80 mg daily was initiated. Hemoglobin improved to 6.4 g/dL on hospital day one with stabilization of hemodynamics reflected by the heart rate of 80-90 beats per minute and blood pressure of 125/85 mmHg. This treatment regimen resulted in rapid clinical improvement, reflected by the recovery of hematologic parameters over the course of four hospital days. The patient was discharged with oral dexamethasone 14 mg daily tapered over a course of six weeks. Iron supplementation was transitioned to ferrous sulfate 325 mg daily, and folic acid supplementation was decreased to 1 mg daily. The patient was agreeable to receive erythropoietin on an as-needed basis.

The patient was referred to rheumatology on discharge and was diagnosed with SLE, thought to be the immune-mediated trigger causing the initial presentation of AIHA and ITP, consistent with secondary ES. Hydroxychloroquine 400 mg daily was initiated. She remained under close surveillance with hematology and rheumatology but unfortunately developed recurrent hemolysis two months after discharge, requiring a pulse dose of oral dexamethasone 40 mg daily for four days. Six months after the initial presentation, routine follow-up labs were again consistent with recurrent hemolysis and thrombocytopenia, requiring the addition of daily oral prednisone 1 mg/kg with a plan to continue low-dose maintenance steroids. Prednisone was maintained at 7 mg daily after an extended taper provided by rheumatology. Ten months after the initial presentation, the patient again developed recurrent hemolytic anemia with hemoglobin of 10.6 g/dL and platelet count dropping to as low as 24,000/µL. Confirmatory DAT IgG and C3 were again positive. Given episodic relapses and significant drops in platelets, prednisone was increased to 50 mg daily and rituximab infusions (800 mg weekly) were initiated for a total of four weekly cycles with a plan to simultaneously wean steroids. Iron studies, folate, and vitamin B12 indices were monitored periodically and replaced. The patient was referred to a tertiary care center for evaluation by hematologists specializing in rare noncancerous blood disorders. She continues to be under close surveillance.

## Discussion

The JW population encompasses at least 7.5 million members worldwide and is one of the most well-known religious communities that decline transfusion of specific blood components based on scriptural interpretation of biblical texts. Practicing JW patients vary in their choice regarding blood product transfusion, and their preference should be established on an individual basis, rather than making assumptions based on prior experiences [5]. Treatment of hematologic abnormalities in JW patients is challenging, regardless of whether aberrant parameters are linked to acute blood loss, bone marrow dysfunction, cellular abnormalities, sequestration, production deficiencies, or autoimmune disorders. Prompt recognition of underlying pathology and clear communication with the patient regarding preferences on transfusion is vital for successful treatment and has been linked to better outcomes [6,7].

Acute symptomatic anemia in the JW population is associated with high mortality rates, especially when hemoglobin levels on presentation are life threatening. Posluszny et al. quote a 2.5-fold increase in mortality for every 1 g/dL drop in hemoglobin less than 8 g/dL based on a retrospective chart review of 300 JW patients presenting with acute anemia [6]. Additionally, multiple retrospective studies have confirmed significantly elevated mortality rates in patients presenting with hemoglobin levels between 5 and 6 g/dL who refuse allogeneic transfusion. Multiple risk stratification models have been employed to calculate mortality rates in JW patients presenting with acute anemia based on the acuity of presentation and risk factors present. These stratification tools are yet to be validated in large multicenter trials but do suggest that mortality rates are significantly elevated in JW patients presenting with anemia [6,7].

Common practices in the treatment of blood cell dyscrasias in patients refusing blood products include the use of ESAs, parenteral iron, vitamin supplementation, phlebotomy minimization, and hemoglobin-based oxygen carriers (HBOCs ) [7]. ESAs are used to induce the proliferation of endogenous erythroid precursors and prevent erythroid cell apoptosis in the bone marrow. Red blood cell (RBC) expansion is evidenced by increased reticulocyte counts within three days of ESA administration; however, a significant treatment response equivalating one unit of packed RBCs can take up to seven days. Prior meta-analyses recommend against the utilization of ESAs in critically ill patients who accept a blood transfusion, given the length of time for recovery in hematologic parameters. However, in the setting of blood product refusal, ESAs are the mainstay of treatment and can potentially be lifesaving [6]. The use of ESAs in ES is limited to small case series and case reports with inconclusive results [1]. This is likely attributed to the rarity of ES, especially in populations that refuse blood transfusion. The use of ESAs in isolated AIHA has been examined in greater detail; however, varying outcomes are described in small multicentric studies [3]. In a single case report, it was theorized that the mechanism of ESAs in AIHA is secondary to both stimulations of RBC production and the ability to decrease the total amount of autoantibodies present on RBCs by increasing the amount of antibody-free erythrocytes in circulation [8]. Additionally, Both TPO-RA and ESAs have been associated with thrombotic events when used in isolated AIHA [1,2].

Parenteral iron is similarly used to ensure adequate iron supply is available to undergo robust erythropoiesis. Iron studies are often skewed in critically ill patients due to the elevation of inflammatory pathways, making it difficult to confirm true iron deficiency. Thus, current protocols utilize varying doses of empiric parenteral iron supplementation in the setting of blood product refusal [6,7]. In one case report, a pregnant patient who developed severe ES was treated empirically with parenteral iron despite normal serum iron studies and demonstrated positive results and recovery of hematologic parameters [9]. While there are anecdotal case reports describing the use of parenteral iron in isolated AIHA and ES, current data are limited and need to be further investigated.

Posluszny et al. describe the usage of HBOCs in the treatment of JW patients presenting with severe anemia, defined by hemoglobin levels less than 5 g/dL or hemoglobin levels of 5-7 g/dL with evidence of hypoperfusion [6]. This alternative form of treatment can be potentially life-saving in the acute setting. Unfortunately, there are multiple limitations, including restricted availability, risk of multiple adverse effects, and minimal supportive data due to lack of clinical trials and multicenter studies. Finally, the minimization of blood draws and diagnostic laboratory testing is crucial to reduce the amount of iatrogenic blood loss, something that was considered in our treatment approach as well [6,7]. The successful treatment of our patient strongly suggests that the strategies listed above can also be utilized in ES, contributing to a scenario that has rarely been described and lacks evidence.

Due to the nature of ES, presentations can vary greatly and patients often face multiple episodes of remissions and exacerbations over the course of their lifetimes, as seen in our patient. Given variable phenotypes and frequent relapses, ES is challenging to treat. Generally considered a diagnosis of exclusion, maintaining a broad differential diagnosis is key to accurately diagnosing and adequately treating the hematologic manifestations of ES. While spontaneous remission has been documented, most patients do require treatment, especially in the acute setting. Although no large-scale randomized controlled studies have been performed to outline a definitive treatment algorithm, most clinicians generally follow a similar approach. First-line therapy focuses on the administration of steroids, with the use of IVIG in severe cases [2,10]. Splenectomy is often utilized in patients presenting with autoimmune cytopenias (AIHA or ITP) who have failed first-line therapies of steroids and IVIG; however, no official recommendations have been made in patients with refractory ES [2]. Currently, second-line therapy focuses on the addition of immunosuppressive agents, such as alemtuzumab, mycophenolate mofetil, cyclosporine, cyclophosphamide, danazol, and rituximab. Of these listed, rituximab has been most frequently used, as it leads to B-cell lymphopenia and reduced autoantibody production. In fact, rituximab has been studied in secondary ES associated with SLE with positive outcomes [1,2]. While the overall mechanism has not been confirmed, the reduction of CD19, CD21, and BR3 on B cells with subsequent reduction in serum Tumour necrosis factor α levels is a proposed theory [11]. Our patient’s clinical course was complicated by a new diagnosis of SLE, requiring a collaborative multidisciplinary approach in the setting of frequent relapses, requiring numerous adjustments to steroid therapy and the eventual addition of rituximab.

Literature on the treatment of ES in the JW population is lacking, likely due to the rarity of ES. While generalized treatment regimens for cytopenias in the JW population have been described as discussed above, the difficulty in treating ES in patients refusing blood products arises with multiple cell line abnormalities and a lack of data available for clinicians. Successful treatment in our patient confirms that the use of ESAs, parenteral iron, minimization of phlebotomy, and vitamin supplementation in addition to steroid therapy can be utilized for JW patients presenting with ES. This case also reiterates the importance of rapid clinical decision-making in JW patients presenting with severe, life-threatening hematologic abnormalities. The relapsing-remitting nature of ES proves to be especially challenging as clinicians often have to treat multiple episodes of severe, recurrent hemolytic anemia and thrombocytopenia, as illustrated by our case.

## Conclusions

Treating blood dyscrasias in the JW population is challenging for medical professionals, especially in the acute setting. It is important to establish each JW patient’s specific preferences regarding blood products. Successful outcomes require rapid recognition of the underlying pathology and creative medical management, as demonstrated by this unique case. Specifically, this report illustrates how the use of ESAs, parenteral iron, and vitamin supplementation can be used to effectively treat ES in practicing JW patients. This blood product-free approach could likely be utilized in multiple other causes of consumptive anemia and thrombocytopenia in patient populations that refuse blood products. Compilation of outcomes is important for the development of targeted recommendations in the treatment of ES and blood product refusal.
